# Supplementary material for: A method to screen for meiotic drive using embryonic markers
Source: G3 (Bethesda). 2026 Sep 23;16(11):jkag242. doi: 10.1093/g3journal/jkag242 (PMC13732436; doi:10.1093/g3journal/jkag242)
Supplement: Supplementary file 1 [file jkag242_supplementary_data.zip › Fig_S1_G3-2026-406919.pdf]

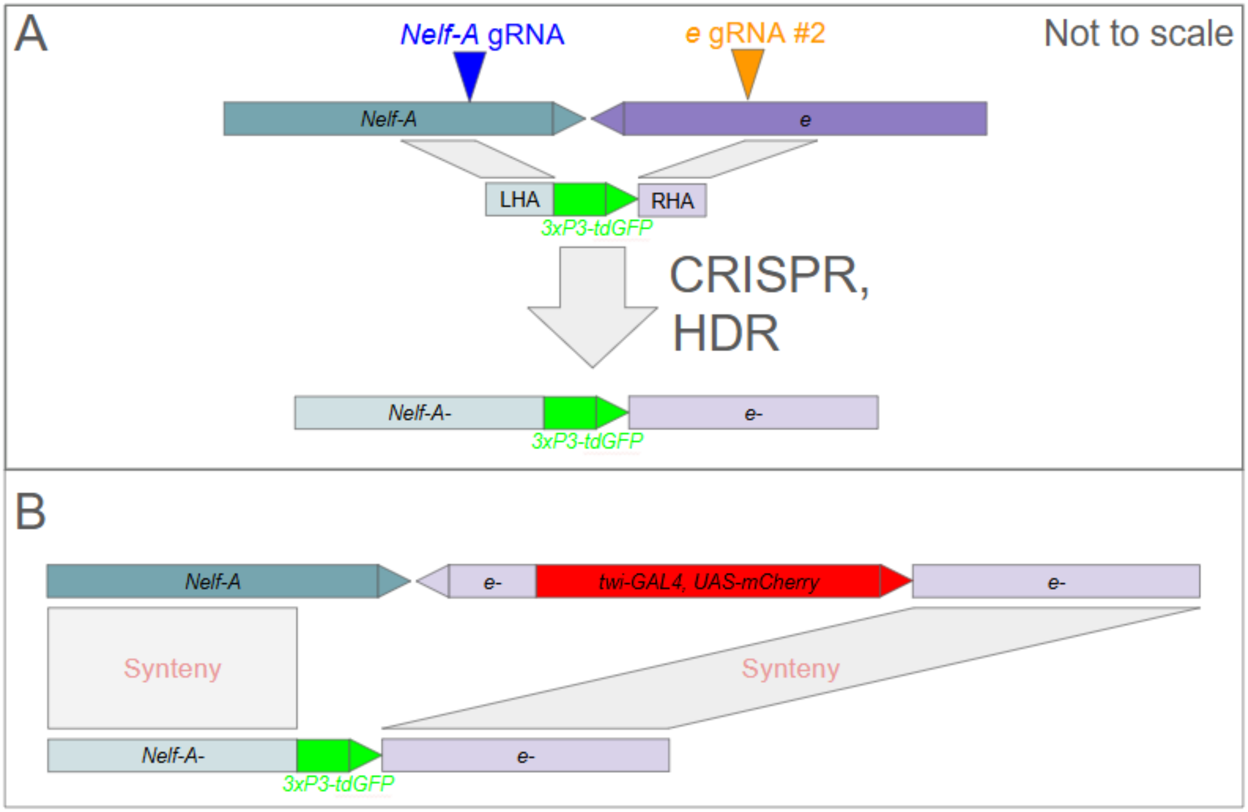

Figure S1: A) Deletion of Nelf-A and replacement with 3xP3-tdGFP via CRISPR; B) Visualization of the genotype of the SPECIES *twi-GAL4, UAS-mCherry/Nelf-A-, e-{3xP3-tdGFP}* stock at the locus where the transgenes are inserted. Because both aberrations are overlapping and homozygous lethal (with no opportunity for recombination to restore full viability to either allele), the alleles “balance” one another, circumventing the difficulty of maintaining the homozygous lethal *twi-GAL4, UAS-mCherry* transgene in the SPECIES background where conventional balancers cannot be introduced.
