# Supplementary material for: A method to screen for meiotic drive using embryonic markers
Source: G3 (Bethesda). 2026 Sep 23;16(11):jkag242. doi: 10.1093/g3journal/jkag242 (PMC13732436; doi:10.1093/g3journal/jkag242)
Supplement: Supplementary file 1 [file jkag242_supplementary_data.zip › Fig_S2_G3-2026-406919.pdf]

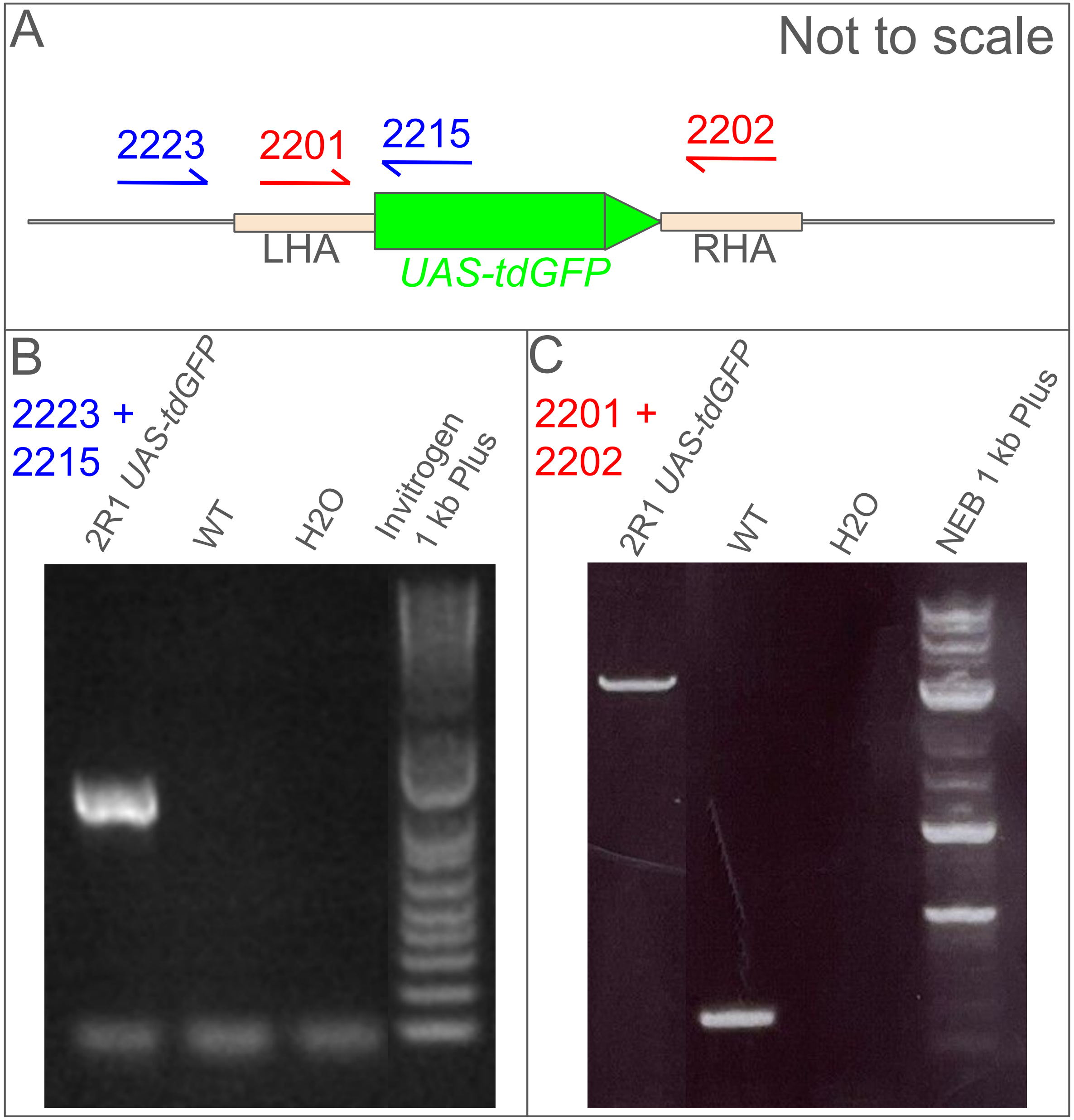

Figure S2: PCR validation confirming proper integration of the *UAS-tdGFP* transgene on chromosome 2; A) Diagram of primer binding sites for genotyping primers relative to the intended transgene insertion; B and C) Results of PCR with primer pairs 2215 + 2223 (B; amplifies a 1.3 kb product only if the transgene is correctly integrated) and 2201 + 2202 (C; amplifies a 3.4 kb product spanning the transgene if it is present, while a 0.2 kb band is produced from uninterrupted wildtype DNA). Note that extra lanes were removed from the images.
