# Supplementary material for: A method to screen for meiotic drive using embryonic markers
Source: G3 (Bethesda). 2026 Sep 23;16(11):jkag242. doi: 10.1093/g3journal/jkag242 (PMC13732436; doi:10.1093/g3journal/jkag242)
Supplement: Supplementary file 1 [file jkag242_supplementary_data.zip › Supplemental_Material_Legends_G3-2026-406919.docx]

- **Supplemental File 1, Table 1:** Raw data underlying Table 1. Counts of embryos and larvae from red fluorescent (RFP^+^), green fluorescent (GFP^+^), and white light (Total, Larvae) images. Note that these categories are not exclusive (i.e. GFP^+^ embryos are a subset of RFP^+^ embryos, which in turn are a subset of the Total). The flies screened for drive were F1s between marker line males and GDL (or SD[72]) females.
- **Supplemental File 1, Table 2:** Sequences of gRNAs and homology arms used for CRISPR mutagenesis in this study.
- **Supplemental File 1, Table 3:** Camera settings used in image acquisition for embryo counting.
- **Supplemental File 1, Table 4:** Primers used to confirm proper integration of transgenes in Drosophila stocks created for this study.
- **Supplemental File 1, Table 5:** Sequences of plasmids injected as part of CRISPR- or phiC31-mediated mutagenesis.
